# Relapsed/Refractory ETP-ALL Successfully Treated With Venetoclax and Nelarabine as a Bridge to Allogeneic Stem Cell Transplant

**DOI:** 10.1097/HS9.0000000000000379

**Published:** 2020-06-08

**Authors:** Ashley McEwan, Omali Pitiyarachchi, Nicholas Viiala

**Affiliations:** 1 Department of Hematology, Liverpool Hospital, Sydney, Australia; 2 Chris O’Brien Lifehouse, Department of Medical Oncology, Sydney, Australia; 3 University of New South Wales, Sydney, Australia

T-lymphoblastic leukemia/ lymphoma (T-ALL/LBL) is a malignant neoplasm of immature T-cells with characteristic immunophenotypic subtypes that correspond to T-cell maturation stages. Early T-cell precursor lymphoblastic leukemia (ETP-ALL) is one such subtype, which is derived from thymic cells at the early T-cell precursor differentiation stage and make up 5% to 16% of T-ALL cases.^1^ ETP-ALL cells are derived from hematopoietic stem cells that have recently migrated to the thymus, and retain multilineage pluripotency.^2^ They display reduced expression of T cell antigens; typically CD1a(−), CD5(−/dim) and CD8(−), and display aberrant expression of stem cell or myeloid antigens.^3^ Congruently, the genetic mutations seen in ETP-ALL are similar to those found in poorly differentiated myeloid neoplasms and mixed phenotype T/myeloid acute leukemia.^4^ Historically, ETP-ALL has a poor prognosis with standard chemotherapy but similar outcomes to T-ALL are reported when treated with contemporary protocols.^3^ Recent research has described how ETP-ALL both expresses and is dependent on BCL2, and hence how antagonists such as ABT-199 (venetoclax) may offer a potential therapeutic benefit.^5^ We report a patient with relapsed/ refractory ETP-ALL, with defined molecular mutations on next generation sequencing (NGS) at the time of relapse, who had an excellent response to venetoclax in combination with nelarabine allowing progression to allogeneic stem cell transplant. We propose this novel treatment strategy is worth further investigation, especially in light of adverse outcomes seen in this disease.

In this report, we describe a case of relapsed/refractory ETP-ALL that responded to the combination of venetoclax and nelarabine, when administered as fourth line therapy. The patient remains in remission over 12 months following sibling allogeneic stem cell transplant.

A 42-year-old woman was diagnosed with ETP-ALL following axillary lymph node biopsy in April 2016. She presented with mild thrombocytopenia and generalized lymphadenopathy in the absence of constitutional symptoms. Biopsy findings were consistent with ETP-ALL (as shown in Fig. 1), with an immunophenotype expressing CD2, CD7, CD34, cytoplasmic CD3, CD13 and negative for surface CD3, CD1a, CD4, CD8, MPO and TdT. FDG-PET/CT at diagnosis showed stage 3 disease with widespread intense FDG-uptake. Less than 1% of BM cells were of the same phenotype as the lymph node biopsy. BM cytogenetic analysis revealed a germ line mutation with a balanced translocation between chromosome 15q22 and 21q22 in all cells, confirmed on subsequent evaluation of peripheral blood lymphocytes. This is clinically insignificant in carriers and without evidence of association with ETP-ALL. Baseline investigations also detected chronic hepatitis B infection with viremia and tenofovir, as treatment and ongoing prophylaxis, was commenced prior to chemotherapy.

**Figure 1:**
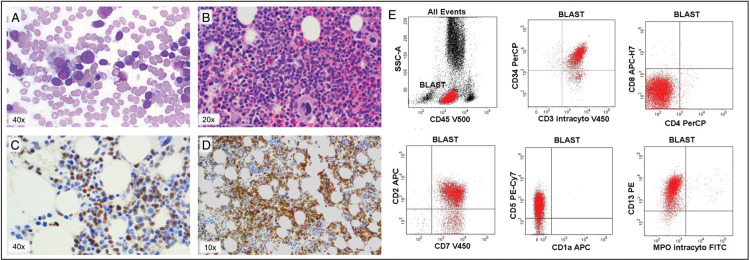
**Bone marrow aspirate and trephine 3/1/18 at initial relapse**. A) Aspirate demonstrating 50% lymphoblasts, B) Haematoxylin and eosin stain on trephine, C) TdT stain on trephine, positive in lymphoblasts, D) BCL-2 stain on trephine, positive in lymphoblasts (A–D) Images taken with an Olympus BX53 microscope with Luminera InfinityHD camera at magnifications listed. E) Flow cytometry results for bone marrow from 3/1/18 demonstrating positive expression in CD34, CD3 intracytoplasmic, CD2, CD7, CD5 and CD13. Negative expression in CD4, CD8, CD1a and MPO Performed on BD FACSCAnto II flow cytometer.

She commenced first line therapy with hyperCVAD and alternating high dose methotrexate and cytarabine, intended to be an eight-cycle treatment course, but limited to six cycles due to complications with bacterial sepsis, liver function derangement and delayed count recovery. FDG-PET/CT performed after 3 cycles and at the completion of 6 cycles demonstrated complete metabolic response. Maintenance therapy with POMP was then commenced, but our patient relapsed during her 14th cycle.

Her relapse was characterized by enlarging mediastinal lymph nodes, intensely FDG avid on PET/CT and correlating to involved nodes at diagnosis. Relapse was confirmed by lymph node biopsy and repeat bone marrow biopsy now showed extensive infiltration with 50% involvement with ETP-ALL (shown in Fig. 1), consistent with the diagnostic immunophenotype (Fig. 1). DNA from this bone marrow sample was later extracted to perform NGS analysis.

Second-line chemotherapy was commenced in January 2018 with FLAG-Ida, as per institutional guidelines, achieving a partial response with bone marrow biopsy detecting 5% blasts on morphology, and 3% ETP-ALL on flow cytometry. FDG-PET/CT imaging post FLAG-Ida demonstrated small volume residual disease within the scapula and clavicle but no uptake in lymph nodes. An inflammatory pulmonary nodule was noted, concerning for possible invasive fungal infection, and for which she received voriconazole therapy. However, in the interval between clearance of infection, acceptance for transplantation, and her conditioning regimen, her disease progressed, with pre-transplant marrow showing 11% lymphoblasts on flow cytometry.

Single agent nelarabine was commenced as third-line chemotherapy utilizing the protocol described by Berg et al.^6^ After 1 cycle of nelarabine, 10% ETP-ALL remained within the bone marrow, which was deemed an insufficient response to proceed on to stem cell transplant. After overt progression of lymphadenopathy, she was subsequently treated with palliative intent with a further cycle of nelarabine, 2 months after her first cycle. Her adenopathy responded partially to the second cycle of nelarabine, and bone marrow biopsy detected 14% ETP-ALL.

In the absence of curative therapeutic options, the patient was offered a novel approach of venetoclax in addition to nelarabine therapy 1 month later in August 2018. Venetoclax was commenced with her third cycle of nelarabine escalating the dose from 200 mg to 400 mg daily, over 3 days. The regimen was well tolerated with no tumor lysis or significant neurotoxicity but moderate cytopenias requiring transfusion support. Posaconazole was added from Day +14, while tenofovir prophylaxis continued throughout. Bone marrow biopsy performed on Day +25 of venetoclax and nelarabine demonstrated morphological complete remission without detectable ETP-ALL by flow cytometry, although no MRD testing was performed. Clinically, there was complete resolution of all lymphadenopathy, but FDG-PET/CT showed abnormal low level uptake in two normal sized cervical lymph nodes and moderate residual focus in the manubrium, a prior site of disease, but possibly reflecting residual bone changes as opposed to ETP-ALL. She continued on venetoclax 400 mg daily for a total duration of 2 months. In October 2018, given doubt over the PET scan, radiation therapy was administered to the sternum, and was followed by conditioning with Cyclophosphamide/Total Body Irradiation, and Sibling Allogeneic Transplant. She remains in remission over 12 months following transplant.

To our knowledge this is one of the first cases of venetoclax used in combination with nelarabine to achieve remission in a patient with relapsed and refractory ETP-ALL. Nelarabine is a deoxyguanosine analog which results in a cytotoxic effect against T lymphoblasts. It is a well-recognized salvage therapy for relapsed/refractory T-ALL albeit resulting in a dismal OS in the absence of Allogeneic Stem Cell Transplant (54% OS with allogeneic-SCT v 22% without).^7^ In this report, the ETP subset was not delineated. Of notable concern is reported cases of irreversible nelarabine neurotoxicity, fortunately not experienced by our patient.^8^

In our patient, Venetoclax, a BH3 mimetic, which inhibits the anti-apoptotic protein BCL-2, was added to a standard salvage regimen of nelarabine. BCL-2 shows high expression in early T-cell precursors and gradually decreases during normal T-cell differentiation. Hence the degree of response to venetoclax may be mediated by the stage of differentiation arrest of the leukemic T cells.^9^ The patient discussed had a significant expression of BCL-2 (Fig. 1), which has been shown to correlate with response to venetoclax.^9^ Several reports have demonstrated the efficacy of venetoclax in combination with other cytotoxic therapies in the setting of relapsed/refractory ETP-ALL and with nelarabine.^10,11^ Despite poor response to single agent nelarabine, our patient was treated with the combination of venetoclax and nelarabine due to the potential synergistic effects of cytotoxicity to T lymphoblasts combined with BCL-2 inhibition, and as an extension of the current widely accepted treatment for relapsed T-ALL. There are several clinical trials currently underway to investigate the use of venetoclax with cytotoxic chemotherapy for the treatment of T-ALL.^12^

Retrospectively, genetic variant data was obtained from DNA extraction, and subsequent next generation sequencing performed on archived bone marrow slides to highlight the distinct genetic profile of ETP-ALL (shown in Table 1). Mutations were detected in the RAS signalling pathway (missense mutations in both JAK1 and JAK3) which have been reported by Zhang et al.^13^ in 67% of ETP-ALL compared to 19% of non-ETP-ALL.

**Table 1:**
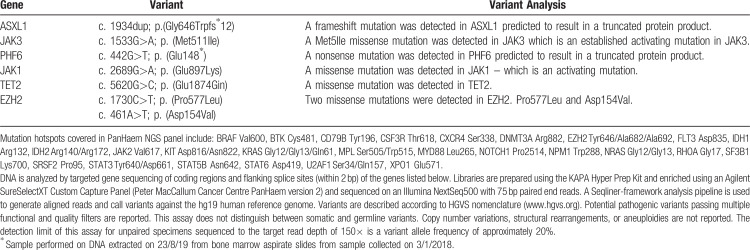
Variants Detected in our Patient on PanHaem NGS Panel^∗^ (Peter MacCallum Cancer Centre)

EZH2 mutations have been shown to increase RAS pathway-associated transcription, potentially creating a transcriptional environment susceptible to other genetic mutations, increasing proliferation.^14^ Two missense mutations in EZH2 were detected which affect histone modification, whom Braggio^14^ reported in 42% of ETP-ALL cases compared with 12% of non-ETP-ALL cases.

A nonsense mutation in PFH6 was also detected. PHF6 mutations have been characterized as a potential early driver mutation in the pathogenesis of T-ALL, as it is implicated in the self-renewal and homeostasis of hemopoietic stem cells.^15^ Lastly, frameshift mutation in ASXL1 and missense mutation in TET2 were detected which affect epigenetic regulation of gene expression. These are classically seen in myeloid disorders but reported at low frequency in T-ALL with TET 2 mutations predominantly in thymic subtypes of T-ALL.^13,14^ This pattern of mutations reflects how the molecular profile of ETP-ALL has a similar pattern to leukemic stem cells and granulocyte precursors and hence retains myeloid potential.^14^ Genetic mutations, immunophenotypic profile and BCL-2 expression detected in ETP-ALL cases inform the basis of alternative treatment strategies based with therapies such as BCL-2 antagonists.

This is one of the first reports of using Venetoclax in combination with Nelarabine as a bridge to transplant in the setting of relapsed/refractory ETP-ALL. She remains in complete remission over 12 months following transplantation. This combination is promising and requires further evaluation as potential salvage therapy in this difficult to treat disease.

## Acknowledgements

The authors thank the Liverpool Hospital clinical flow cytometry scientist Neil McNamara, for help with flow cytometry data.
